# Effect of chlorhexidine on the durability of a new universal adhesive system

**DOI:** 10.4317/jced.53794

**Published:** 2018-09-01

**Authors:** Niloofar Shadman, Shahram Farzin-Ebrahimi, Elaheh Mortazavi-Lahijani, Zahra Jalali

**Affiliations:** 1 DDS, MSc, Associate Professor, Department of Operative Dentistry, School of Dentistry, Kerman University of Medical Sciences, Kerman, Iran; 2 DDS, MSc, Assistant Professor, Department of Operative Dentistry, School of Dentistry, Kerman University of Medical Sciences, Kerman, Iran; 3 DDS, MSc Student, Department of Operative Dentistry, School of Dentistry, Kerman University of Medical Sciences, Kerman, Iran

## Abstract

**Background:**

The effect of chlorhexidine on bond durability of universal adhesives is not clear. The aim of this study was to evaluate the effect of chlorhexidine on 6-month water storage bond strength of adhesive systems.

**Material and Methods:**

72 freshly sound human extracted molars were selected. In each tooth both buccal and lingual sides were prepared by bur to reach superficial dentin and randomly divided into 6 groups and 12 sub-groups and bonded with Scotchbond Universal (SBU) or Scotchbond Multi-purpose (SBMP) with/without chlorhexidine (CHX) usage. Group 1: SBU, group2: SBU+CHX, group3: Etch+SBU, group4: Etch+CHX+SBU, group5: Etch+SBMP, group6: Etch+CHX+SBMP. After composite curing, water storage and thermocycling was done. Each group was divided into two subgroups. One was tested immediately, and the other was thermocycled for 5000 cycles (5-55 °C) (it was equivalent to 6 months of storage in distilled water). Shear bond strength test was done and failure modes were determined by Stereomicroscope. The data were analyzed by one-way ANOVA, Tukey post-hoc test and Paired Two test with *P*<0.050 as the level of significance.

**Results:**

Shear bond strength in late SBU (Self etch) was significantly lower than late SBU [Etch and rinse (ER)], P value= 0.0001, also shear bond strength in late SBU [self-etch (SE)] was significantly lower than immediate SBU (SE), P value= 0.01. There were no significant differences between other sub-groups and conditions. The most failure mode was adhesive in all the groups.

**Conclusions:**

Long term bonding durability of SBU(ER) was better than SBU (SE). CHX usage had prevented bond strength decrease in SBU and SBMP in long term. CHX usage did not have any effect on immediate shear bond strength of SBU and SBMP. Immediate and late shear bond strength of SBMP with/without CHX usage was similar to SBU(SE, ER).

** Key words:**Dentin Bonding, Shear Bond Strength, chlorhexidine, 6-month storage.

## Introduction

Good adhesion is required for a successful composite restoration. There is a challenge in bonding to dentine because of dentinal fluid, variable tubular structure, and high organic content (1). Composite restoration longevity depends on hybrid layer quality and integrity. Recent studies have shown that during acid etching, matrix metalo proteinase enzymes (MMPs) release from dentine and can degrade hybrid layer. Using MMP inhibitors such as Glardin, tetracyclin, hypochlorite, green tea, or chlorhexidine (CHX) is an approach for preventing hybrid layer degradation (2-4). There is contrary about the effect of CHX on bond strength (immediate or delayed) of composite to dentin. Sinha *et al.* were demonstrated that CHX application had significantly increased immediate bond strength (5).In Gunaydin *et al.* study it was concluded that CHX was reduced immediate bond strength in self-etch and etch-and-rinse adhesives but after 6 month( 5000 cycles) in CHX treated groups, bond strength was higher (6). Previous literatures have demonstrated that dentine bonding durability of etch-and-rinse adhesives can be improved by applying CHX before hybrid layer formation. In the other hand, there are controversial studies about the CHX effect on protecting bonding stability in self-etch adhesives (7). Nano leakage can happen in etch-and-rinse adhesives because of discrepancies between demineralization depth and resin infiltration depth (8) which is less in self-etch adhesives since demineralization and resin penetration can occur simultaneously (9). One bottle self-etch adhesives are so hydrophilic that cause water sorption and dentine-adhesive interface degradation is happen after long time water storage. Some manufactures produce a one bottle adhesive which can be used both methods (etch-and-rinse and self-etch). They are called multi-purpose, multi-mode or universal adhesives. Universal Scotchbond (SBU) is one of them which contain water, alcohol, HEMA, Vitrebond copolymer, MDP acidic monomers and silane. SBU shows high bond strength in both of modes (8,10). The aim of this study was evaluating the effect of 2 % chlorhexidine on immediate and 6-month storage shear bond strength of SBU in two different methods of dentine conditioning: etch-and-rinse and self-etch. The null hypotheses were 1. There is no significant difference between shear bond strength of SBU in different etching modes and SBMP, 2. 6-month storage has no effect on SBU and SBMP bond strength, 3. Using CHX has no effect on SBU and SBMP bond strength in immediate and 6-month storage status.

## Material and Methods

**Figure 1:**
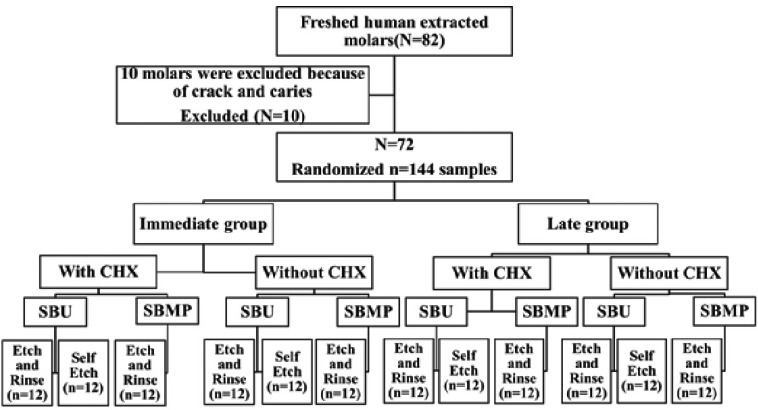
Flow diagram consort.

**Figure 2:**
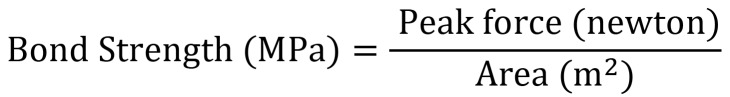
Equation.

This *in vitro* study was done on 72 freshly human extracted third molar teeth without any crack and caries (Fig. 1). After removing calculus and soft tissue, they were stored in disinfectant solution for 24 hour and then in distilled water In each tooth both buccal and lingual sides were used for bonding (number of samples=144). The mid surfaces of buccal and lingual surfaces were prepared in 1.5 mm depth with a fissure diamond bur (teezkavan, Iran) to reach the superficial dentin. The flat dentin surfaces were polished with 600-grit silicon carbide abrasive paper (Matador, Germany) to provide a standardized smear layer. Teeth were mounted up to the cementoenamel junction in the self-cure acrylic resin (Acropars,Iran) in a way that the occlusal surfaces of the teeth were located horizontally. Teeth were randomly divided into 6 groups and 12 sub-groups (throw a dice, below and equal to 3 – immediate groups, over 3 – late groups). Adhesives used in this study were Scotchbond Universal (3M, ESPE, USA), (SBU), and Scotchbond Multi-purpose, (3M, ESPE, USA), (SBMP). Group 1: SBU, group2: SBU+CHX, group3: Etch+SBU, group4: Etch+CHX+SBU, group5: Etch+SBMP, group6: Etch+CHX+SBMP. 2% chlorhexidine digluconate solution (Consepsis, Ultradent, USA) was applied on the dentin surface prior to application of adhesives and gently air dried. All of the materials were used according to manufacturer’s instructions ( Table 1). After adhesives application, curing was done by a LED light curing unit (Elipar, 3M, and ESPE) with 600 mW/cm2 intensity. Then, the microhybrid composite (Filtek Z-250 XT, Shade: B1,3M, ESPE, USA) was placed on the bonded area by a clear plastic cylindrical tube (2 mm diameter and 2 mm height) in two layers and each layer was cured for 20 seconds. After removing the tubes, samples were stored in distilled water in an incubator at 37 °C for 24 hours then were thermocycled for 500 cycles (5-55 °C).Then each group was divided into two subgroups. One of the subgroups was tested immediately, and the other sub-group was thermocycled for 5000 cycles (5-55 °C) (it was equivalent to 6 months of storage in distilled water). The shear bond strength test (blade type) was done by the Universal Testing Machine (Testometric M350-10 CT, Lancashire, United Kingdom) with 0.5 mm/min crosshead speed with a chisel-shaped device. The shear bond strength was calculated in megapascal (MPa) by the below equation: (Fig. 2).

**Table 1:**
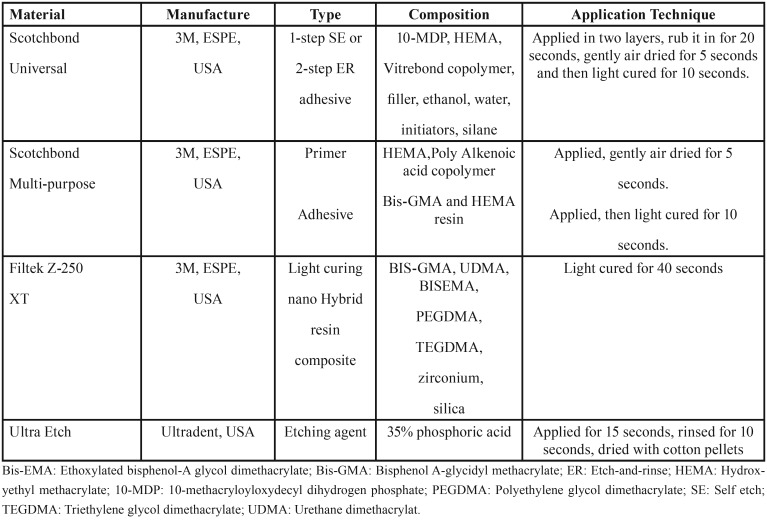
Composition and application techniques of the tested materials.

Mode of failure was identified by two examiners by observed the deboned surface levels separately by a stereomicroscope (Olympus, DP12, Germany) at × 40 magnifications. Finally, the type of failure (cohesive in composite, cohesive in dentine, adhesive or mixed-partially adhesive and partially cohesive) was identified and recorded on the agreement of observers. To compare the shear bond strength in each group one-way ANOVA analysis and post-hoc test [Tukey HSD (honest significant difference)] and two compare groups together Paired Two test was used. *P* ˂ 0.05 was set as the level of significance.

## Results

Shear bond strength in late SBU(SE) was significantly lower than late SBU(ER), (*P* value= 0.0001), also shear bond strength in late SBU(SE) was significantly lower than immediate SBU(SE), *P* value= 0.01. There were no significant differences between other sub-groups and conditions. The most failure mode was adhesive in all the groups ( Table 2).

**Table 2:**
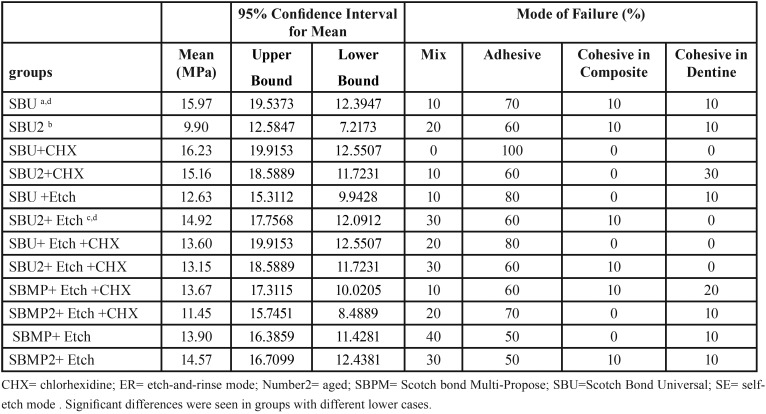
Shear bond strength data in MPa and mean percentage of failure mode.

## Discussion

The results of the present study showed that there is no significant difference between SBU(SE) and SBU(ER), except late shear bond strength (SBS) between SBU(SE) and SBU(ER); so the first null hypothesis was partially confirmed.

Perdiago *et al.* in a study showed that there was no significant difference between micro tensile bond strength (µTBS) of SBU in different etching modes. High bond strength in SE mode is probably due to the presence of 10-MDP in SBU adhesive. This monomer can form Ca-10-MDP which is a stable salt. Chemical bond between polycarboxylic monomers and hydroxyapatite can increase bond strength (11).

In the present study two layer of SBU adhesive (in SE mode) was used like Munoz *et al.* study (12). It seems that the extra layer of adhesive can compensate monomers inefficiency in penetrating into smear layer and dentin in this self-etch adhesive. Polymerization was improved by increasing adhesive thickness. In the present study, adhesive was rubbed on dentin surface for 20 seconds; this resulted in better monomer penetration. This bonding improvement can be a rationalization for the difference between SBS in SE and ER mode.

Acid etching can improve interface morphology by forming a thick hybrid layer and long resin tags. Smear layer removing can result in a more convenient adhesive penetration. Perdiago *et al.* indicated that inadequate polymerization due to oxygen inhibition in thin adhesive layers is the reason of lower bond strength in all in one adhesives (11).

Takamizava *et al.* did not find any significant difference between SBS in different etching modes for universal adhesives (13). For SBU these results were similar to the result of this study. They reported that SBU has clinically acceptable bond strength and this does not seem to change with different etching modes (SE or ER). They also said that SBU contains 10-MDP and Vitrebond which cause bonding to hydroxyapatite and bringing high bond strength for SE mode. There is little clinical literature on universal adhesive bond stability; these studies concluded that universal adhesive′s bond stability is clinically accepted in both SE and ER modes (13).

In the present study there was no significant difference between immediate and late SBS of SBU(ER) and SBMP, except SBU (SE). The second null hypothesis was partially approved. The lower thickness of hybrid layer may be the cause of this difference.

Wagner *et al.* thermocycled their specimens for 5000 cycles. They concluded that acid etching application does not affect universal adhesive bond strength after thermocycling (14). Lnoue s *et al.* also showed that there was no reduction after 100000 thermal cycles in tensile bond strength for 10-MDP containing adhesives (15). It can be concluded that for interface destruction of 10-MDP containing adhesives like SBU, 100000 thermal cycles or long term water storage is required and it is probably the reason of our results which showed no reduction in bond strength of ER mode after thermocycling.

Base of the result of our study, CHX had no effect on both etching mode of SBU and SBMP, in comparison of immediate and late bond strength, this indicates a positive effect of CHX in preserving bond strength in short and long term, so the third hypotheses was confirmed.

Campos *et al.* reported that CHX as a MMP inhibitor reduces the resin-dentin interface destruction and prevents the late bond strength reduction. They also reported that application of CHX 2% has an adverse effect on SE adhesive bond strength such as ClearFil SE bond and ClearFil Tri S bond, and it should be prevented before SE adhesives. CHX and adhesive component interaction may cause lesser wettability and dentin conditioning by some SE adhesives. This controversy may be because of different experimental methods, different test design and various spectrums of tested materials (16).

Zheng *et al.* concluded that CHX could prevent µTBS reduction after aging in ER systems, but did not have any effect on SE adhesives. These results were partially similar to our results. Effect of CHX is probably related to adhesive type and maybe there is not a general rule about this (17).

Shafiei *et al.* concluded that CHX could reduce the loss of bond strength of ClearFil protect bond and Clear SE bond adhesives over time, but it had an adverse effect on immediate bond strength. These results could be related to CHX preserving effect on bonding interface (18). The benefit of CHX 2% on hybrid layer stability (after aging) of ER and SE adhesives was shown in this study.

SBU can make a strong bond to enamel and dentin in its both etching modes because of the presence of 10-MDP and Vitrebond in its composition. SBMP also have Vitrebond.

Komori *et al.* did not find any significant difference in SBMP bond strength to dentin with and without CHX application after 6 month storage in artificial saliva (19). These findings were similar to ours. Clinical performance of 3-step ER adhesives is good comparing to simplified ER adhesives. Presence of an insoluble hydrophobic layer on hybridized dentin can improve dentine sealing by reduction of permeability in resin-dentin interface (19).

Studies about CHX application effect on bond strength in different adhesives are controversial (7,20).

In the present study the most failure mode was adhesive. Failure mode in SBS tests is because of stress distribution during force load and does not necessarily show bond performance.

Takamizava *et al.* showed that the most failure mode in SBU was cohesive in dentin (13).

In Munoz *et al.* (21) and Perdiago *et al.* (11) studies, the most failure mode was adhesive, which was like our study.

Different etching modes for universal adhesives produce various weak areas in the adjacent interface between adhesive layer and resin composite or between decalcified dentin and adhesive layer. Furthermore, such areas and flawed bonding may adversely influence long-term bonding durability.

## Conclusions

With the limitation of this study, it was concluded that long term bonding durability of SBU(ER) was better than SBU (SE). CHX usage had prevented bond strength decrease in SBU and SBMP in long term. CHX usage did not have any effect on immediate shear bond strength of SBU and SBMP. Immediate and late shear bond strength of SBMP with/without CHX usage was similar to SBU (SE, ER).
